# Structure of putative epidermal sensory receptors in an acoel flatworm, *Praesagittifera naikaiensis*

**DOI:** 10.1007/s00441-024-03865-y

**Published:** 2024-02-02

**Authors:** Tosuke Sakagami, Kaho Watanabe, Mayuko Hamada, Tatsuya Sakamoto, Toshimitsu Hatabu, Motonori Ando

**Affiliations:** 1 Laboratory of Animal Physiology and Pharmacology, Department of Animal Science, Graduate School of Environmental and Life Science, Okayama University, Okayama, 700-8530 Japan; 2 Laboratory of Cell Physiology, Department of Science Education, Graduate School of Education, Okayama University, Okayama, 700-8530 Japan; 3 Ushimado Marine Institute, Graduate School of Natural Science and Technology, Okayama University, Okayama, 701-4303 Japan

**Keywords:** Phalloidin, α-Tubulin, dSap47, Polycystin, Xenacoelomorpha

## Abstract

Acoel flatworms possess epidermal sensory-receptor cells on their body surfaces and exhibit behavioral repertoires such as geotaxis and phototaxis. Acoel epidermal sensory receptors should be mechanical and/or chemical receptors; however, the mechanisms of their sensory reception have not been elucidated. We examined the three-dimensional relationship between epidermal sensory receptors and their innervation in an acoel flatworm, *Praesagittifera naikaiensis*. The distribution of the sensory receptors was different between the ventral and dorsal sides of worms. The nervous system was mainly composed of a peripheral nerve net, an anterior brain, and three pairs of longitudinal nerve cords. The nerve net was located closer to the body surface than the brain and the nerve cords. The sensory receptors have neural connections with the nerve net in the entire body of worms. We identified five homologs of polycystic kidney disease (*PKD*): *PKD1-1*, *PKD1-2*, *PKD1-3*, *PKD1-4*, and, *PKD2*, from the *P. naikaiensis* genome. All of these *PKD* genes were implied to be expressed in the epidermal sensory receptors of *P. naikaiensis*. *PKD1-1* and *PKD2* were dispersed across the entire body of worms. *PKD1-2*, *PKD1-3*, and *PKD1-4* were expressed in the anterior region of worms. *PKD1-4* was also expressed around the mouth opening. Our results indicated that *P. naikaiensis* possessed several types of epidermal sensory receptors to convert various environmental stimuli into electrical signals via the PKD channels and transmit the signals to afferent nerve and/or effector cells.

## Introduction

Acoela are aquatic bilateral flatworms with a simple body plan. They lack gut epithelium, anus, and coelomic cavity. Their simplicity is suggested to represent ancient bilaterian characteristics or secondary losses from a complex ancestor because Acoela belong to the phylum Xenacoelomorpha which forms a sister group to Nephrozoa (Cannon et al. 2016) or Ambulacraria (Marlétaz et al. 2019; Mulhair et al. 2021; Philippe et al. 2019). Some of them exhibit behavioral repertoires such as geotaxis (Keeble 1912; Sakagami et al. 2021; Sprecher et al. 2015), phototaxis (Keeble 1912; Nissen et al. 2015; Sprecher et al. 2015; Yamasu 1991), and rheotaxis (Keeble 1912). To perceive environmental stimuli, they possess epidermal sensory-receptor cells, one statocyst, and paired eyes. The epidermal sensory receptors are located on their entire body surface and should be mechano- and/or chemo-receptors (Bedini et al. 1973). Several types of them have been identified from some acoel species (Todt and Tyler 2007; Bery et al. 2010). The statocyst and eyes are located in the anterior region of worms and are gravity and photoreceptors, respectively (Sprecher et al. 2015; Sakagami et al. 2021; Yamasu 1991).

These sensory organs should be innervated. Acoel nervous systems are mainly composed of an anterior brain, three to five pairs of longitudinal nerve cords, and a peripheral nerve net. The brain, surrounding both the statocyst and eyes at the anterior region of worms, has been described by various terms such as the cerebral ganglion (Ferrero 1973), commissural brain (Raikova et al. 1998), and statocyst ganglion (Bery et al. 2010). The nerve cords extend from the brain along the entire length of worm bodies (Achatz and Martinez 2012; Raikova et al. 1998; Sakagami et al. 2021; Sprecher et al. 2015). The nerve net is a peripheral neural plexus with the shape of a network (Bedini and Lanfranchi 1991; Bery et al. 2010). Acoel epidermal sensory receptors are bipolar and multipolar neurons. So far, ultrastructural analyses of the epidermal sensory receptors have revealed that their dendrites carry a sensory cilium which is responsible for receiving environmental stimuli, that their cell bodies are sunk underneath the epidermis, and that their axons form synapses with other neurons (Arboleda et al. 2018; Bery et al. 2010; Martinez et al. 2017, 2021; Pfistermüller and Tyler 2002; Rieger et al. 1991). Transcriptome analyses have identified some transient receptor potential (TRP) channels that should be involved in acoel sensory functions (Duruz et al. 2021; Hulett et al. 2022). However, signal transduction mechanisms in acoel epidermal sensory receptors have not been elucidated.

In this study, we used *Praesagittifera naikaiensis* which is a Japanese endemic acoel species (Hikosaka-Katayama et al. 2020; Yamasu 1982), because it has a ciliated epidermis with epidermal sensory receptors (Sakagami et al. 2021) and we can access the genome data of *P. naikaiensis* (Arimoto et al. 2019). To examine the relationship between the epidermal sensory receptors and their innervation, we simultaneously visualized both of them. Acoel epidermal sensory receptors are classified into collared or non-collared receptors characterized by the presence or absence of a microvillar collar surrounding a single sensory cilium (Bedini et al. 1973; Pfistermüller and Tyler 2002; Rieger et al. 1991; Semmler et al. 2008; Todt and Tyler 2007; Zabotin 2019). Since cilia and microvilli are composed of microtubules and actin filaments, respectively, we used α-tubulin antibody and fluorescent phalloidin. A neural marker was used, *Drosophila* synaptic protein 47 (dSap47) antibody, that selectively labels nerve terminals (Reichmuth et al. 1995; Sakagami et al. 2021; Sprecher et al. 2015).

To reveal signal transduction molecules in acoel epidermal sensory receptors, we focused on the polycystin family which is required for mechano- and/or chemoreception in other organisms such as the Cnidaria *Hydra magnipapillata* (McLaughlin 2017), the Nematoda *Caenorhabditis elegans* (Barr 2005; Barr and Sternberg 1999), and the Annelida *Platynereis dumerilii* (Bezares-Calderón et al. 2020)*.* The polycystin family is divided into two types of channel subunits, polycystic kidney disease (PKD) 1-related subunits and PKD2-related subunits, which are the member of TRP polycystin (TRPP) family (Esarte Palomero et al. 2023). We identified five *P. naikaiensis* homologs of the polycystin family and showed that these genes could be expressed in the epidermal sensory receptors. To the best of our knowledge, this study is the first report to identify molecules that might be required for mechano- and/or chemo-reception in acoel epidermal sensory receptors.

## Materials and methods

### Animals

*P. naikaiensis* were collected in the upper part of the intertidal zone on the Seto Inland Sea coasts in Japan. They were kept in aquariums with filtered seawater at 18 °C with a 12:12 h light-dark cycle. The filtered seawater was changed weekly or according to needs. Worms were harvested within 1 month of collection. All worms were immersed in 7.14% MgCl_2_ hexahydrate to avoid muscle contraction before fixation. Using ImageJ software (National Institutes of Health, Bethesda, Maryland, USA), we measured the length of sensory cilia. All data were expressed as means ± SE (*n*, number of worms).

### Scanning electron microscopy

Worms were fixed in 4% paraformaldehyde and 0.1 M phosphate buffer (pH 7.4) for 30 min at room temperature. After washing with 0.1 M phosphate buffer, the worms were dehydrated in a graded series of ethanol concentrations, were critical point dried, and then sputter-coated with osmium. Using a scanning electron microscope (S4800, Hitachi, Tokyo, Japan) at 20 kV, the specimens were examined.

### Transmission electron microscopy

Worms were immersed in a fixative containing 2.5% glutaraldehyde and 0.1 M cacodylate buffer (CB) containing 6% sucrose (pH 7.4) for 90 min at room temperature. After washing with 0.1 M CB, the worms were post-fixed in 1% OsO_4_ and 0.1 M CB for 90 min at room temperature. After washing with 0.1 M CB, the worms were dehydrated in a graded series of ethanol concentrations, transferred through propylene oxide, and then embedded in Spurr’s resin. Using a transmission electron microscope (H-7650, Hitachi, Tokyo, Japan) at 80 kV, thin sections were made and stained with uranyl acetate and lead citrate and examined.

### Immunohistochemistry and confocal laser microscopy

Immunohistochemical analysis was performed as previously described (Sakagami et al. 2021). The following primary antibodies were used: mouse anti-dSap4 antibody (DSAP47-1, Developmental Studies Hybridoma Bank, Iowa, USA) at a 1:20 dilution and mouse anti-α-tubulin antibody (DM1A, Thermo Fisher Scientific, Tokyo, Japan) at a 1:200 dilution. The following secondary antibodies were used: goat anti-mouse IgG antibody, Alexa Fluor 488 (A11001, Thermos Fisher Scientific, Tokyo, Japan), at a 1:200 dilution. Cytological staining for microvilli and muscles was done with Acti-stain 555 phalloidin (PHDH1-A, Cytoskeleton Inc., Denver, USA) at a 1:200 dilution. Fluorescent images were taken using a confocal laser scanning microscope system (FV1200, Olympus, Tokyo, Japan). Depth coding, stereo pair, and orthogonal images were reconstructed from confocal image stacks using LSM 510 software (version 3.2; Carl Zeiss, Germany) and Imaris software (version 9.3; Oxford Instruments, Oxfordshire, UK).

### Phylogenetic analysis

Using amino acid sequences of *Homo sapiens* homologs of polycystin family proteins, a similarity search was conducted by the Basic Local Alignment Search Tool for Protein searches against the *P. naikaiensis* genome at https://marinegenomics.oist.jp/p_naikaiensis/viewer?project_id=71 (Arimoto et al. 2019), with an *E*-value cutoff that was less than 1e − 10. A phylogenetic tree was generated using amino acid sequences of PKD proteins from *P. naikaiensis*, *H. sapiens*, *Drosophila melanogaster*, *C. elegans*, and *Nematostella vectensis*. These amino acid sequences of multiple alignments were produced by ClustalX version 2.1 with gap trimming (Larkin et al. 2007). Sequences of poor quality that were not well aligned were deleted using BioEdit (Hall 1999), and then, a phylogenetic tree was constructed using the neighbor-joining (NJ) method by ClustalX. Bootstrap support values were obtained from 1000 replicate resampled data. After that, the phylogenetic tree was drawn by using the NJ plot (Perriere and Gouy 1996). Domain searches against the Pfam database (Pfam-A.hmm) were performed using HMMER (Eddy 1998; Finn et al. 2016). Gene model ID were as follows: *PKD1-1* (g8209), *PKD1-2* (g20157), *PKD1-3* (g14832), *PKD1-4* (g6405), and *PKD2* (g29246).

### RNA extraction and subsequent cDNA synthesis

Total RNA was extracted using a QIA shredder and an RNeasy Mini kit (Qiagen, Tokyo, Japan) and treated with DNase I (Qiagen) digestion to avoid DNA contamination. The quality and quantity of RNA preparations were assessed using a NanoDrop ND-1000 (Thermo Fisher Scientific, Waltham, MA). Isolated RNA was stored at − 80 °C until use. The single-strand cDNA was synthesized using ReverTra Ace reverse transcriptase (Toyobo, Tokyo, Japan) and oligo-dT primers (Toyobo) for 40 min at 42 °C. DNA fragments of *PKD* genes were amplified by PCR with gene-specific primers which were designed with the Primer 3 program at http://primer3.sourceforge.net/ (Table 1).

**Table 1:** Primers used in this study

Gene	Primer sequences (5′-3′)	Size (bp)	Gene model ID
*PKD1-1*	GGGCATTCGTGTCTGGATTTACTG	1031	g8209
	GGTGTTTGTATCGGCAGCGG		
*PKD1-2*	GCGGGCTATCATGCTCGAGTTC	1092	g20157
	GTCTGCCATGTTTCGTCGTCTG		
*PKD1-3*	ATCAGTTCATCCACCGGTCCATAG	1093	g14832
	CTCTCAATCGTTCGGTCAATCAGC		
*PKD1-4*	GCCTTTGAAGATTCTCATCCTGGC	1035	g6405
	GCTATCATGGTCACAACGGCG		
*PKD2*	GTGTCTGCGTTCGACCGATTC	901	g29246
	CTGCAAACATGGCTCTAATCTCCG		

### Whole-mount in situ hybridization

Amplified cDNA fragments were ligated into pGEM-T easy vector (Promega, Madison, WI, USA) and cloned. Gene-specific antisense or sense digoxigenin (DIG)-labeled cRNA probes were synthesized with a Roche DIG RNA labeling kit (Roche Diagnostics, Penzberg, Germany). Worms were fixed in 4% paraformaldehyde and 0.1 M phosphate buffer (pH 7.4) for 20 min at room temperature and washed with phosphate-buffered saline (PBS) containing 0.1% Tween-20. The worms were then dehydrated in a graded series of methanol concentrations and stored at − 30 °C until use. After rehydration washing with 1 × SSC (15 mM Na citrate and 150 mM NaCl and pH adjusted to 7.0), bleaching was carried out with 2% H_2_O_2_ containing 5% formamide and 0.5 × SSC for 90 min at room temperature. The worms were then washed with 1 × SSC, treated with 0.01 mg/ml Proteinase K for 5 min, and followed by 4% paraformaldehyde and 0.1 M phosphate buffer (pH 7.4) for 20 min at room temperature. DIG-labeled cRNA probe hybridization was performed in a solution containing 1% Tween-20, 5% dextran sulfate, 50% formamide, 5 × SSC, and 0.1 mg/ml tRNA from baker’s yeast for overnight at 56 °C. After blocking with 3% normal goat serum and 0.5% blocking reagent (Roche Diagnostics), the worms were incubated with alkaline-phosphatase-conjugated anti-DIG antibody (Roche Diagnostics; 1:10,000 dilution) for overnight at 4 °C and were washed with 50 mM Tris-HCl (pH 7.5) containing 150 mM NaCl and 0.1% Tween-20. The last wash was followed by Tris-HCl (pH 9.5) containing 100 mM NaCl and 50 mM MgCl_2_. The color reaction was developed in 3.5 µl/ml 5-bromo-4-chloro-3-indolyl phosphate toluidine (Roche Diagnostics) and 4.5 µl/ml nitroblue tetrazolium (Roche Diagnostics) at 4 °C. When staining was satisfactory, the worms were washed with PBS containing 0.1% Tween-20 and dehydrated in a graded series of methanol concentrations. After the worms were rehydrated and cleared in 75% glycerol, we observed using a differential interference contrast microscope (IX71, Olympus, Tokyo, Japan) equipped with an Olympus DP21 microscope camera.

## Results

### Structure of epidermal sensory receptors

**Fig. 1:**
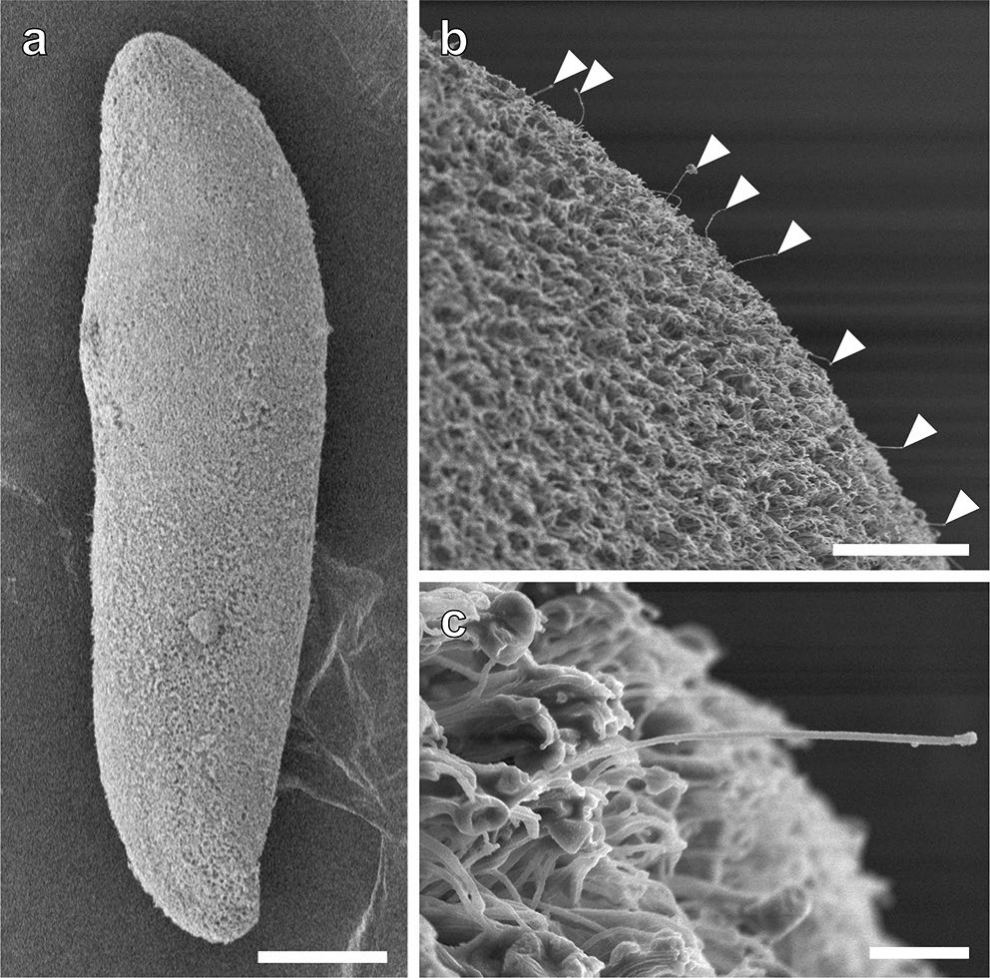
Sensory cilia in the worm body surface. **a** Epidermal cilia covering the entire body surface. Anterior facing upward. **b** Sensory cilia showing longer than epidermal motile cilia (*arrowheads*). **c** A fine structure of the sensory cilium. Scale bars, 150 µm (**a**), 20 µm (**b**), and 2 µm (**c**)

**Fig. 2:**
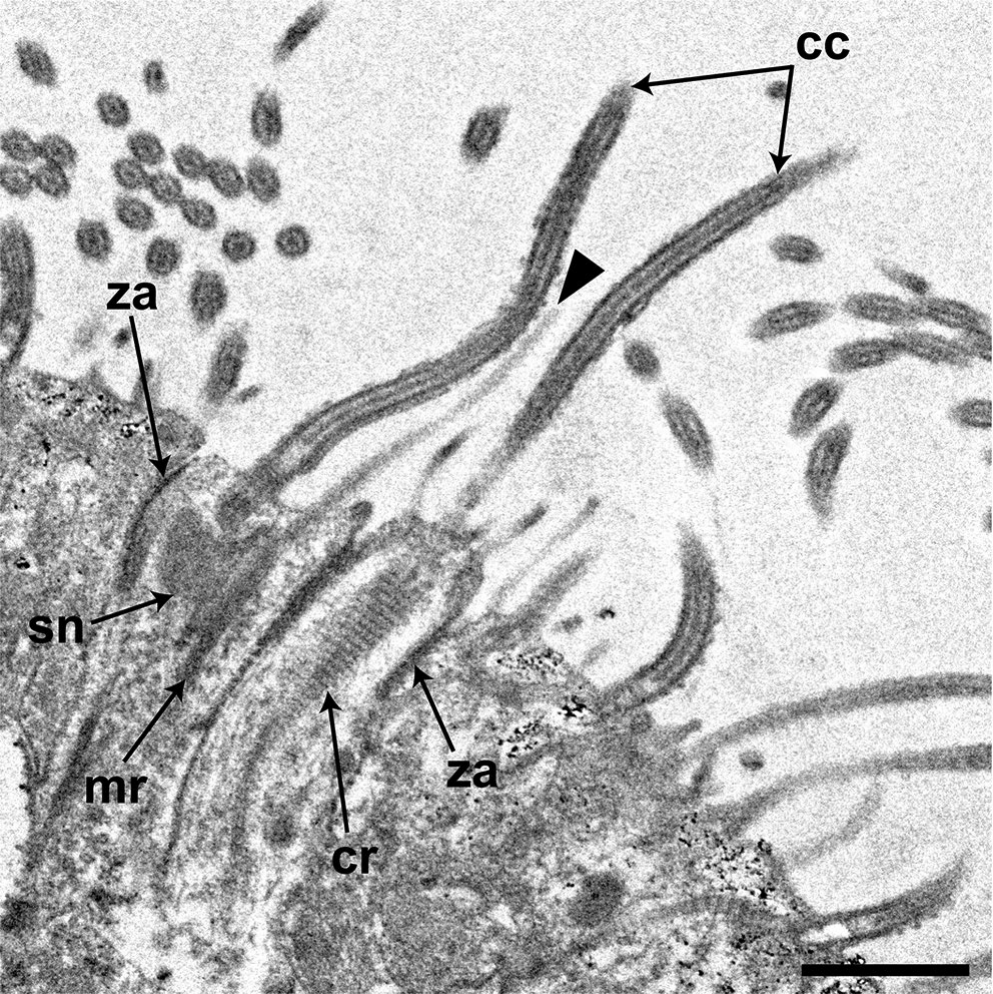
Fine structure of epidermal sensory receptors. *Note*: both collared and non-collared sensory receptors are observed. *Arrowhead*, a microvillus of the collared receptor. *cc* central cilium, *cr* ciliary rootlet, *mr* microvillous rootlet, *sn* swallow’s nest, and *za* zonula adherens. Scale bar, 1 µm

Scanning electron microscopy revealed that the epidermis of *P. naikaiensis* was covered with motile and non-motile ciliated cells (Fig. 1a). The non-motile cilium was a sensory cilium which may be a dendrite of the epidermal sensory receptor (Rieger et al. 1991). The sensory cilia were observed on the entire body surface of worms, but those were denser at the anterior and posterior tips (Fig. 1b, c). Transmission electron microscopy revealed that there were two types of epidermal sensory receptors, collared and non-collared receptors (Fig. 2). The collared one possessed a collar of microvilli surrounding the central cilium and an electron-dense core called a swallow’s nest instead of a rootlet (Pfistermüller and Tyler 2002). The non-collared one possessed a single rootlet. These receptors were connected to the adjoining epidermal cells through a zonula adherens.

**Fig. 3:**
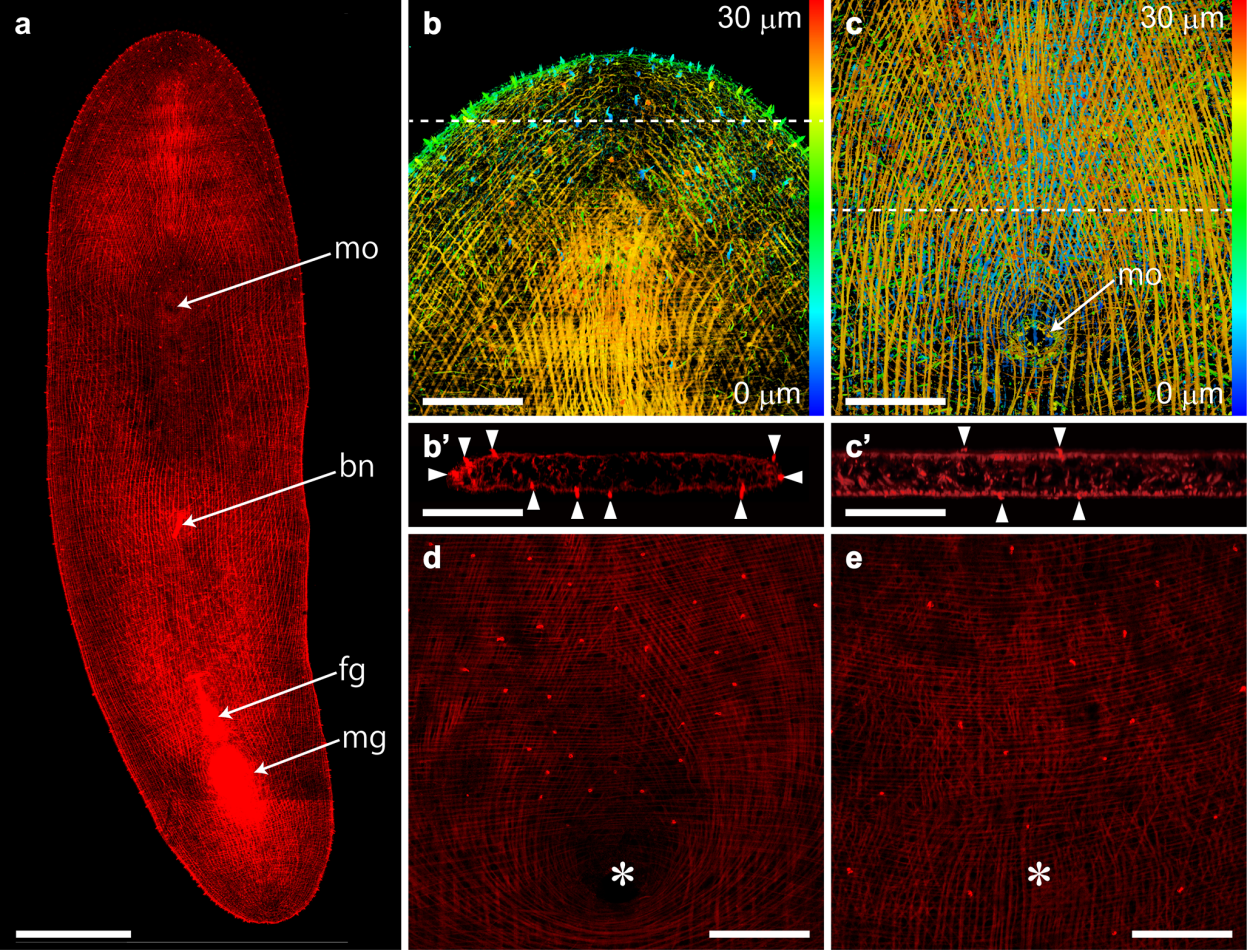
Phalloidin staining showing the structure of actin filaments. **a** A montage image of the whole body was created by 4 projection images. Each projection image was reconstructed from 40 optical sections taken at 0.6 µm. The body-wall musculature of adult worm. Anterior facing upward. **b**, **c** Depth coding images at the anterior (**b**) and middle (**c**) regions of worms. Each image was reconstructed from 150 optical sections taken at 0.2 µm. Color code: *blue* (dorsal) to *red* (ventral). *Dotted lines* indicate the sectioning plane for **b’** and **c’**, respectively. Phalloidin staining showing microvilli of the collared receptors and cross-over muscles. **b’**, **c’** Reconstructed *x–z* images at the anterior (**b’**) and middle (**c’**) regions of worms. Dorsal side facing upward. **d**, **e** Confocal optical sections of the ventral (**d**) and dorsal (**e**) surfaces of the same worm body. *Note*: the collared receptors are concentrated anterior to the mouth opening on the ventral side. *Asterisks* indicate the position of the mouth opening. *Arrowheads,* a microvillus of the collared receptor. *bn* bursal nozzle, *fg* female gonopore, *mg* male gonopore, and *mo* mouth opening. Scale bars, 200 µm (**a**), 50 µm (**b**, **b’**, **c**, **c’**, **d**, and **e**)

Phalloidin staining revealed the distribution of collared sensory receptors and the worm’s musculature (Fig. 3). The collared receptors were observed on the entire body surface of worms and were denser at the anterior and posterior tips (Fig. 3a, b). Their collars showed a brush-like shape and protruded beyond an epidermal cell web which is formed by actin filaments as a cytoskeletal element (Fig. 3b, b’, and c’; Tyler 1984). The collared receptors were also denser anterior to the mouth opening on the ventral side (Fig. 3d) but were scattered on the dorsal side (Fig. 3e).

The musculature of the worm body wall was comprised of three layers of myofibers, including circular, longitudinal, and diagonal muscles (Fig. 3a–c). Circular and longitudinal muscles ran perpendicular and parallel to the anterior–posterior axis, respectively. The longitudinal muscles were located underneath the circular muscles (Fig. 3b, c). Diagonal muscles ran from the anterior lateral sides to the midline until half of the worm body. Some of the diagonal muscles were fused at the mouth opening on the ventral side (Fig. 3c, d). The mouth opening was formed by encircled muscles and was located in the middle region of worms (Fig. 3a, c, d). Additionally, phalloidin staining allowed for the visualization of both male and female gonopores as well as a bursal nozzle (Fig. 3a). These genital organs were not observed in newly hatched juvenile worms (data not shown).

**Fig. 4:**
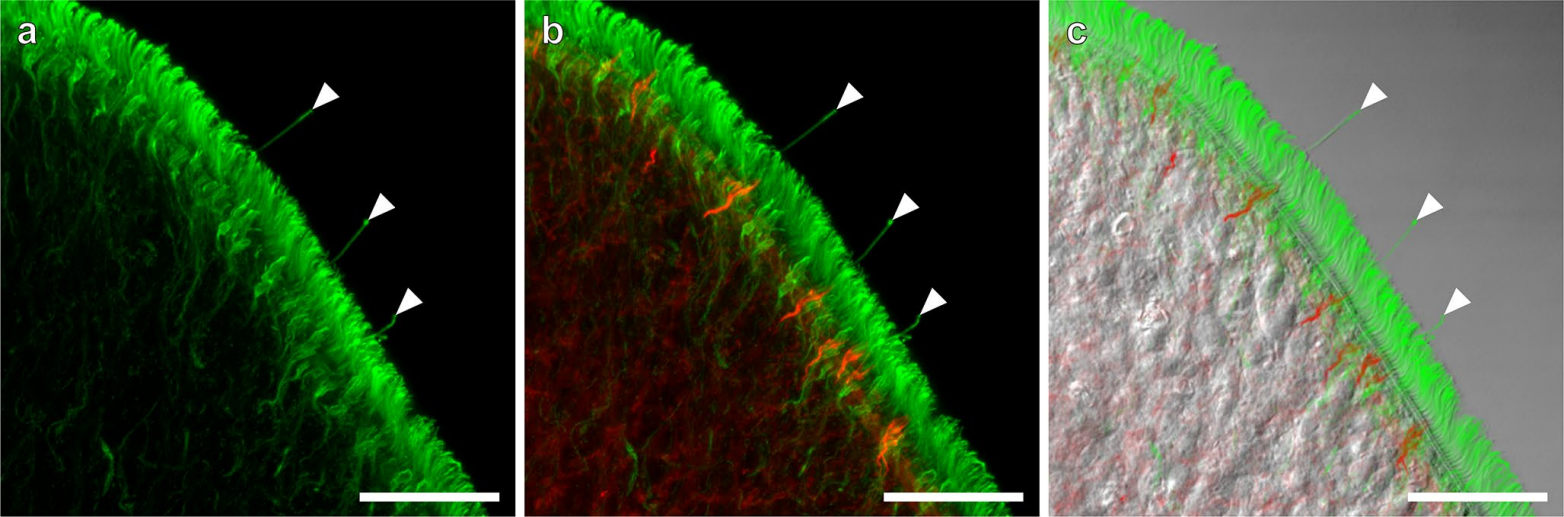
Sensory cilia and microvilli of collared receptors. **a** Sensory cilia were labeled by the α-tubulin antibody. **b** Double labeling for sensory cilia (*green*) and microvilli (*red*) of collared receptors. **c** A transmission light micrograph overlaid with *green* and *red* fluorescence. Projection images were reconstructed from 76 optical sections taken at 0.05 µm. *Note*: the collared receptor composed a single cilium and microvilli. *Arrowheads*, sensory cilia. Scale bars, 20 µm

**Fig. 5:**
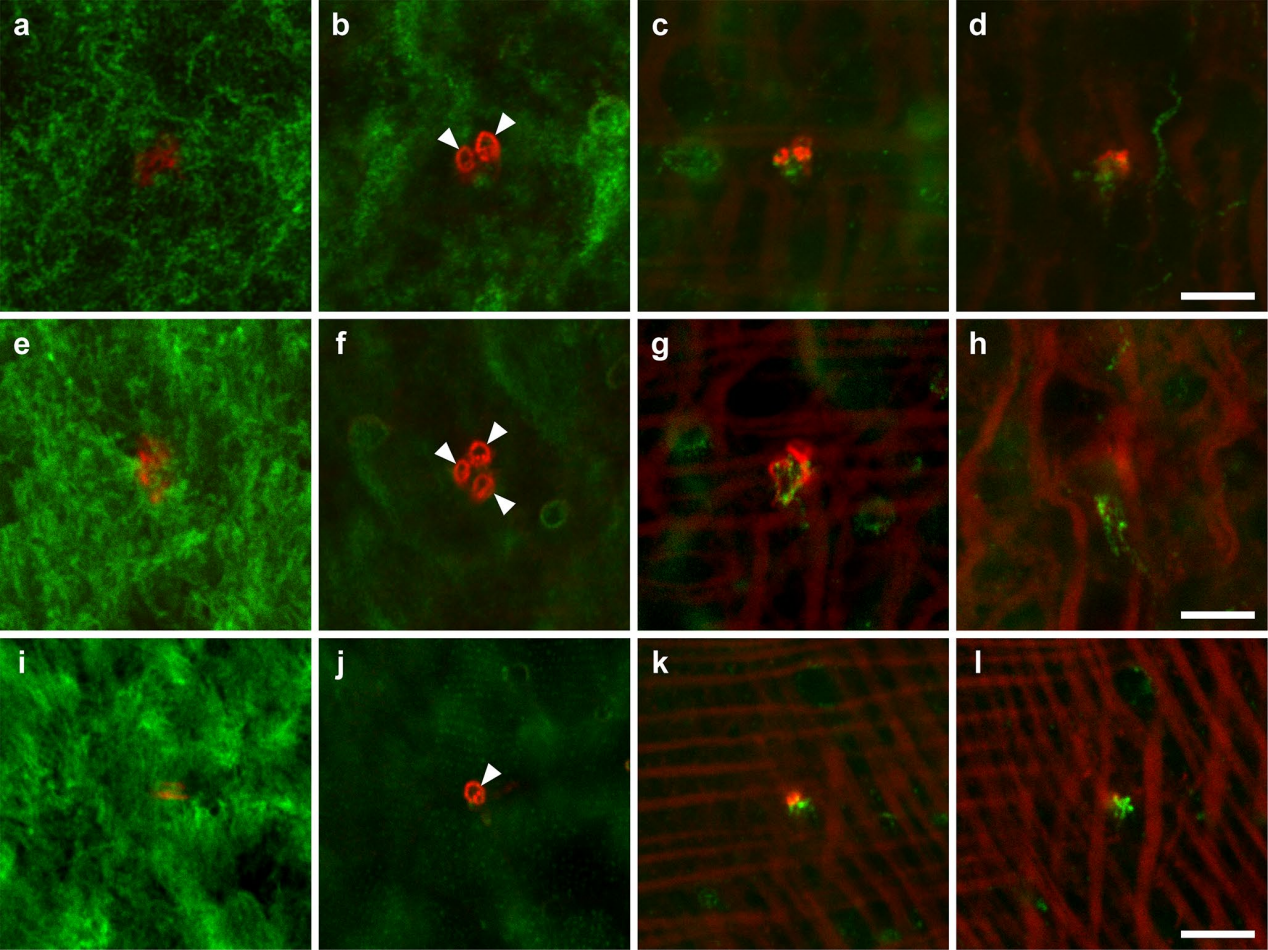
Top views of collared receptors. Serial confocal optical sections were viewed from epidermal cilia to underlying muscle layers. Sensory cilia and microvilli of collared receptors were labeled by the α-tubulin antibody (*green*) and fluorescent phalloidin (*red*), respectively. **a**–**h** Clusters formed by two (**a**–**d**) and three (**e**–**h**) collared receptors on the dorsal surface. **i**–**l** A single collared receptor on the ventral surface. *Arrowheads*, the collar of microvilli. Approximate depths from the epidermal ciliary surface were at 1 µm (**a**, **e**, **i**), 1.5 µm (**b**, **f**, **j**), 2 µm (**c**, **g**, **k**), and 2.5 µm (**d**, **h**, **l**), respectively. Scale bars, 5 µm

**Fig. 6:**
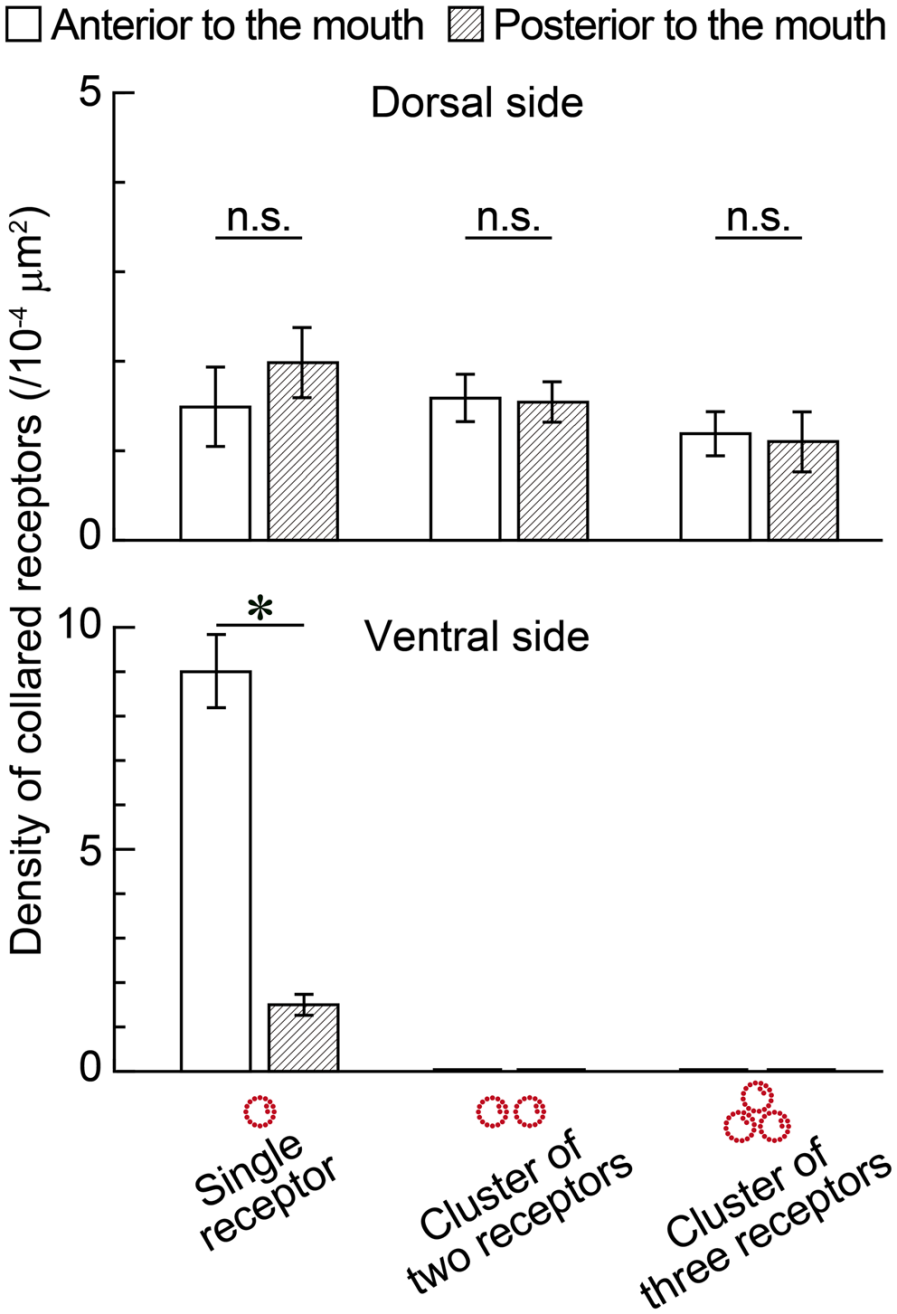
Density of collared receptors. The number of the collared receptors was counted in 100-µm squares at the anterior (*open bar*) and posterior (*hatched bar*) to the mouth opening on the dorsal and ventral sides, respectively. Data are expressed as mean ± SE (*n* = 10). *Asterisk* indicates a statistically significant difference (*P* < 0.01). *n.s*. no significant difference

Immunopositive patterns of α-tubulin visualized epidermal cilia including the epidermal sensory receptors (Fig. 4). The length of sensory cilia was 14.7 ± 2.1 µm (*n* = 5). These cilia were observed on the entire body surface of worms and were denser at the anterior and posterior tips, as were observed by scanning electron microscopy (Fig. 1). The distribution of some sensory cilia corresponded to those of the microvillus collars which were observed below the sensory cilia (Figs. 4 and 5). There were some clusters formed by two or three collared receptors on the dorsal side (Fig. 5a–h). Such clusters were not observed on the ventral side (Fig. 5i–l). Several microvilli were encircled by the collars (Fig. 5b, f, j, *arrowheads*). Actin filaments of these microvilli extended to a level of their cell bodies and connected one another (Fig. 5c, d, g, h, k, and l). Axons were also visible at a level below the cell bodies (Fig. 5c, d, g, h, k, and l). The density of collared receptors visualized with phalloidin was measured on both the dorsal and ventral sides (Fig. 6). There was no significant difference in the density between the anterior and posterior regions to the mouth opening on the dorsal side. In contrast, the density showed a significant difference on the ventral side.

### Relationship between epidermal sensory receptors and their innervation

**Fig. 7:**
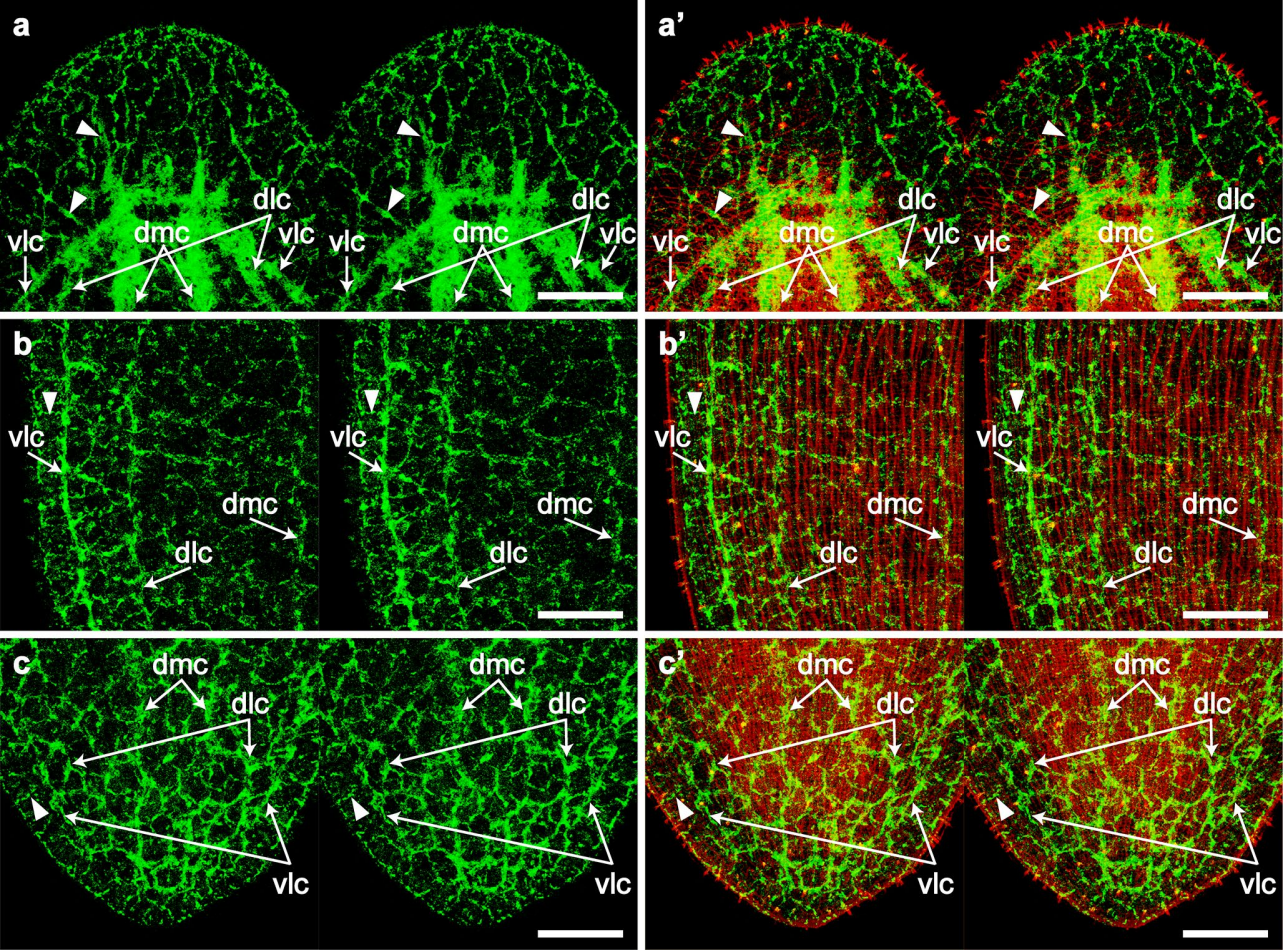
Three-dimensional relationship between the nervous system and collared receptors. The nervous system and microvilli of collared receptors were labeled by the dSap47 antibody (*green*) and fluorescent phalloidin (*red*), respectively. **a**, **a’** Anterior region. **b**, **b’** Lateral region. **c**, **c’** Posterior region. Stereo pairs of confocal images were reconstructed from 171 optical sections taken at 0.2 µm. To obtain stereo images, view the left panel with the left eye and the right panel with the right eye at a distance of 30–35 cm. Each image is viewed from the dorsal side. *dmc* dorsomedial nerve cord, *dlc* dorsolateral nerve cord, and *vlc* ventrolateral nerve cord. *Arrowheads* indicate neurites extended from the peripheral nerve net to the brain or nerve cords. Scale bars, 20 µm

Immunopositive patterns of dSap47 revealed the relationships between the collared receptors and their innervation in the anterior, lateral, and posterior regions of worms (Fig. 7a–c), and we could observe them three-dimensionally with confocal microscopy. The nervous system was mainly composed of a peripheral nerve net, an anterior brain, and three pairs of longitudinal nerve cords. Among these, the nerve net was located closer to the body surface. There were neural connections between the collared receptors and the nerve net. Some neurites extended from the part of the nerve net to the brain at the anterior region (Fig. 7a, a’) and to the nerve cords at the lateral and posterior regions (Fig. 7b, b’, c, and c’). We also observed some neurites interconnected among the collared receptors.

### Expression and localization of *PKD* genes in *P. naikaiensis*

**Fig. 8:**
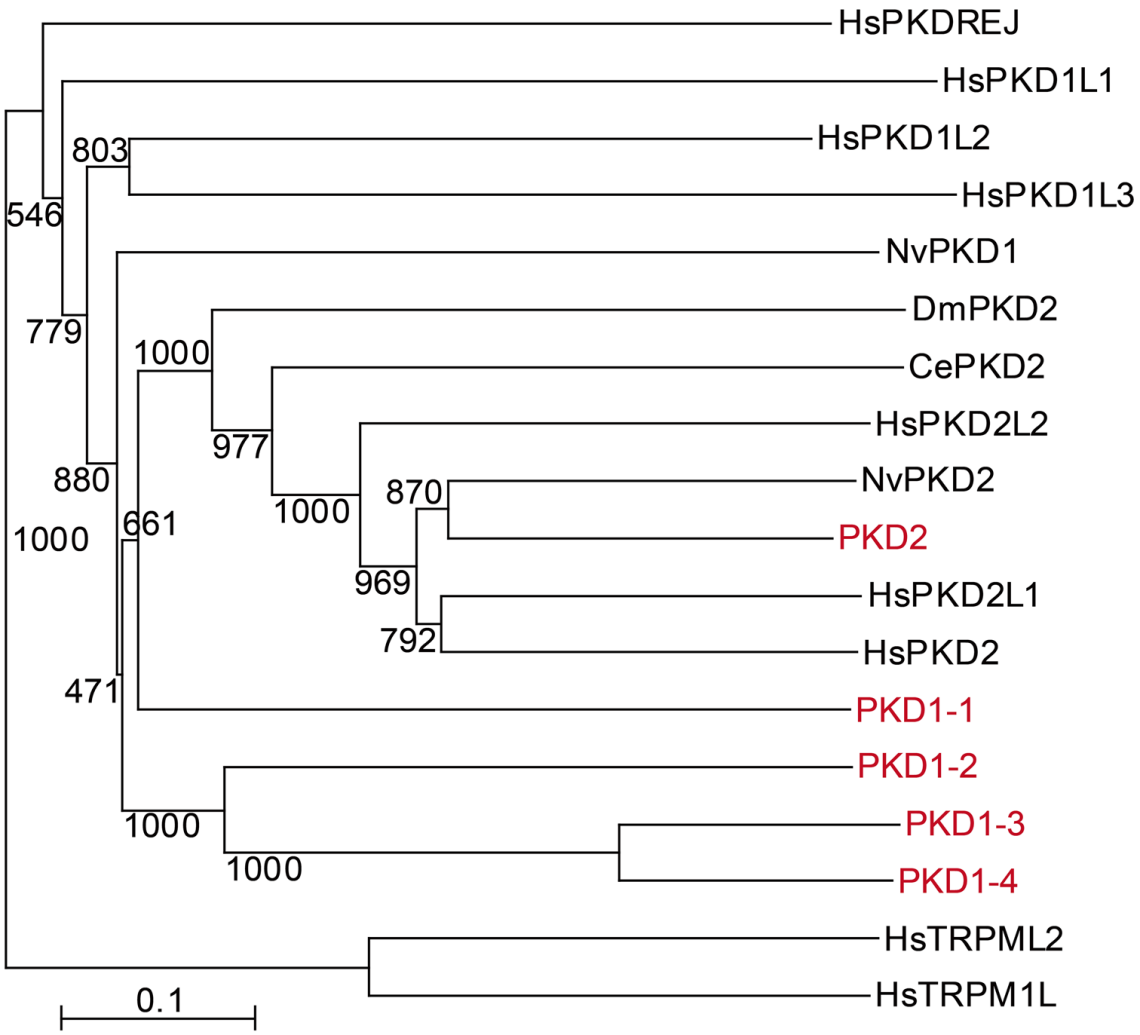
Phylogenetic analysis of the *PKD* homologs. The phylogenetic tree was depicted by neighbor-joining method and was rooted by TRPML family genes in *H. sapiens*. The values of replicate trees in which the associated taxa clustered together in the bootstrap test (1000 replicates) is shown next to the branches. The tree is drawn to scale, with branch lengths in the same units as those of the evolutionary distances used to infer the phylogenetic tree. Red, *Praesagittifera naikaiensis*; black, *Caenorhabditis elegans* (Ce), *Drosophila melanogaster* (Dm), *Homo sapiens* (Hs), and *Nematostella vectensis* (Nv)

BLAST hits and phylogenetic reconstruction methods revealed five candidate genes on the genome of *P. naikaiensis*, with high similarity to known polycystin family genes (Fig. 8). We named them *PKD1-1*, *PKD1-2*, *PKD1-3*, *PKD1-4*, and *PKD2*. Among these, *PKD2* showed high similarity to known PKD2-related subunits of other bilaterians (Fig. 8). All of the *PKD* genes contained Polycystin_dom (PF20519.1) and PKD_channel (PF08016.15) Pfam domains which were found in PKD1-related and PKD2-related proteins of *H. sapiens*. *PKD1-1*, *PKD1-2*, *PKD1-3*, and *PKD1-4* contained PLAT (PF01477.26) Pfam domain which was found in PKD1-related proteins of *H. sapiens*.

**Fig. 9:**
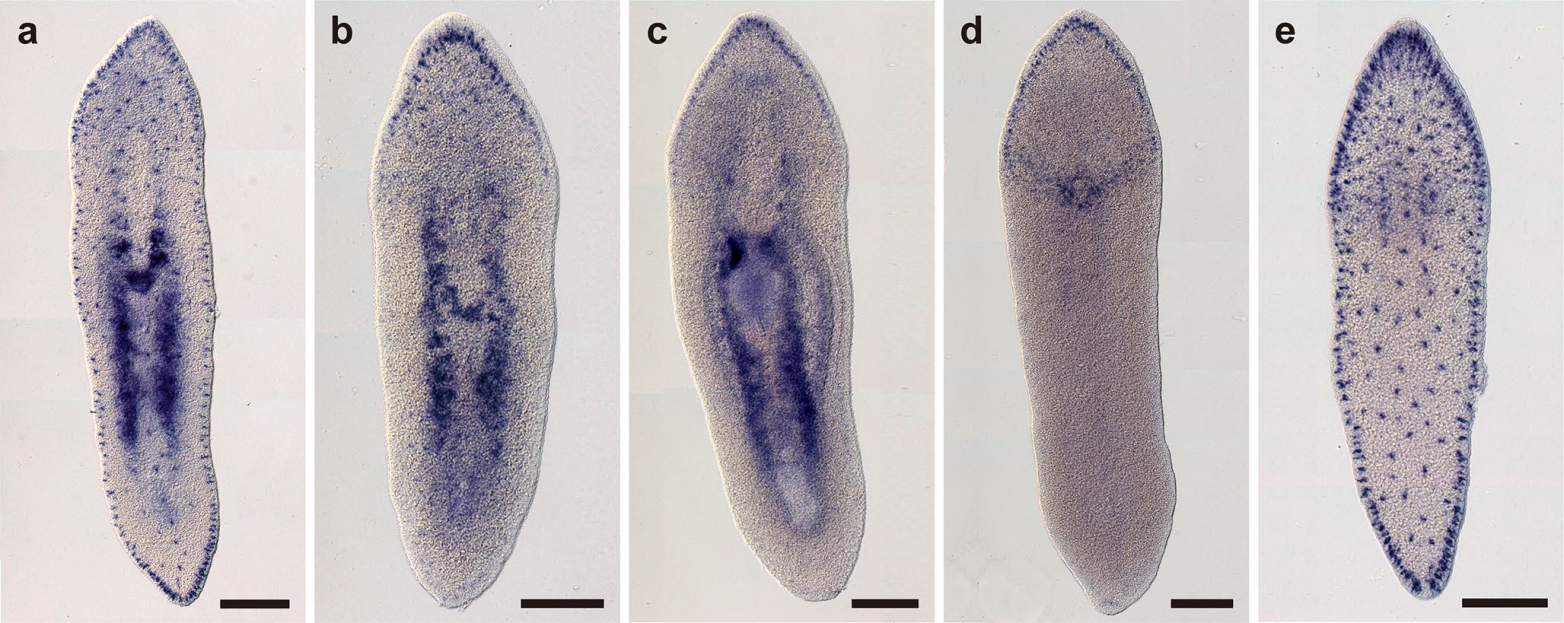
Gene expression patterns of the *PKD* homologs of *P. naikaiensis*. **a** *PKD1-1*. **b** *PKD1-2*. **c** *PKD1-3*. **d** *PKD1-4*. **e** *PKD2*. Montage images of the whole body were created by 5 transmission light micrographs. Scale bars: 150 µm

Whole-mount in situ hybridization revealed that all of the *PKD* genes were expressed in cells which located in the peripheral region (Fig. 9). The cell bodies were sunk underneath the epidermis, implying that these cells were the epidermal sensory receptors. *PKD1-1* and *PKD2* were dispersed across the entire body of worms (Fig. 9a, e). These were highly expressed in the anterior and posterior regions compared with the lateral region (Fig. 9a, e). *PKD1-2*, *PKD1-3*, and *PKD1-4* were expressed in the anterior region (Fig. 9b–d). *PKD1-4* was also expressed around the mouth opening (Fig. 9d). In the region of testes, *PKD1-1*, *PKD1-2*, and *PKD1-3* were expressed (Fig. 9a–c). No signals were seen in the sense controls (data not shown).

## Discussion

Phalloidin staining could visualize collared receptors, which has been reported in the Convolutidae *Symsagittifera roscoffensis* (Semmler et al. 2008), in the Isodiametridae *Isodiametra pulchra* (Pfistermüller and Tyler 2002; Tyler and Rieger 1999), and in the Proporidae *Proporus bermudensis* (Todt and Tyler 2007). The term “swallow’s nest receptors” refers to these collared receptors. Their actin filaments of microvilli extend and connect one another at a level of their cell bodies. Instead of striated rootlets, these receptors consist of an electron-dense core called a swallow’s nest (Pfistermüller and Tyler 2002; Todt and Tyler 2007). Additionally, these receptors are found in a more derived acoel clade named Crucimusculata (Jondelius et al. 2011) including the Convolutidae (Bedini et al. 1973; Bery et al. 2010), the Isodiametridae (Pfistermüller and Tyler 2002), and the Proporidae (Todt and Tyler 2007). Because *P. naikaiensis* belong to the Convolutidae (Jondelius et al. 2011) and their collared receptors visualized with phalloidin showed similar structures of actin filaments to the swallow’s nest receptors, the collared receptor of *P. naikaiensis* was suggested to be homologous to them.

Distributions of epidermal sensory receptors have been investigated in several acoel species. A cluster of sensory receptors has been reported in Acoela (Bery et al. 2010; Todt and Tyler 2007); however, that of the swallow’s nest receptors has not yet been done. *P. naikaiensis* did display clusters of collared receptors on the dorsal side, suggesting acoel swallow’s nest receptors form such clusters. Additionally, there is a possibility that the phalloidin staining visualizes another collared receptor that possesses a ciliary rootlet splitting into numerous striated fibers (Todt and Tyler 2007). Until now, the collared receptor with the striated rootlet has been observed in the Convolutidae *S. roscoffensis*, *C. convoluta*, and *Convoluta theca*, and that of *C. convoluta* forms a cluster on the dorsal side (Bery et al. 2010; Todt and Tyler 2007). Even though this collared receptor has not been reported to visualize with fluorescent phalloidin, our findings suggested that there were several types of collared receptors on the dorsal side of *P. naikaiensis*. Some platyhelminths possess various sensory receptors on the dorsal sides to detect mechanical, chemical, and visual stimuli (Cribb et al. 2003; Watson and Rohde 1994; Whittington et al. 1999). The dorsal side of *P. naikaiensis* might be capable of detecting a wide range of stimuli as well. Future studies are required to reveal the role of acoel epidermal sensory receptors by electrophysiological analysis.

Epidermal sensory receptors should be innervated and transmit environmental stimuli to afferent neurons. So far, ultrastructural analyses have revealed that epidermal sensory receptors of *S. roscoffensis* form synapses with neurons and effector cells such as muscles and glands (Arboleda et al. 2018; Martinez et al. 2021). Such neural connections between the collared receptors and the nerve net were observed three-dimensionally on the entire body of *P. naikaiensis*. Acoel flatworms exhibit a body contraction response induced by mechanical and chemical stimuli (Sprecher et al. 2015; Tyler and Rieger 1999). *S. roscoffensis* exhibits the body contraction response even after the amputation of its head, which includes the brain (Sprecher et al. 2015). We also observed the same response in *P. naikaiensis* after the amputation of its head (data not shown). Our results corroborated Sprecher et al.’s (2015) theory that the behavior was brain-independent and that they could sense things via epidermal mechanical and/or chemical sensors connected to nerve cords.

To convert the mechanical and/or chemical stimuli into electrical signals in epidermal sensory receptors, acoel flatworms should possess signal transduction molecules such as PKD channels. Among five *PKD* homologs identified from the *P. naikaiensis* genome, *PKD2* was shown to be classified into the PKD2-related subunits and other *PKD* genes appeared to show species-specific gene duplication. In a mammalian kidney, PKD1-like and PKD2-like proteins act as a heteromeric channel and are found in primary cilia which can reversibly bend in response to fluid flow rates (Praetorius and Spring 2003a, b). In the renal tube, bending of the primary cilium by the shear flow leads to activating PKD1L1/PKD2 channels (McGrath et al. 2003; Nonaka et al. 2002). Therefore, in *P. naikaiensis*, PKD1-1 and PKD2, as well as PKD1-2 and PKD1-3, may form heteromeric channels, respectively. Additionally, it is possible that each *P. naikaiensis* PKD either forms a heteromeric channel with other TRPs or a homomeric channel. In human Madin-Darby canine kidney cells, a heteromeric channel of PKD2 and TRPV4 forms a complex that is both mechanical and thermal receptors (Köttgen et al. 2008).

Acoel flatworms exhibit behavioral repertoires which may be required for *PKD* genes. The body contraction response in *P. naikaiensis* was induced by simple mechanical stimulation (e.g., tapping the Petri dish) even after the amputation of its head (data not shown), suggesting water-borne vibration activates PKD1-1 and/or PKD2 channels because these were expressed entire body surface. Besides, acoel flatworms detect water flows, warm temperatures, and chemicals. *S. roscoffensis* show rheotaxis and thermotaxis (Gamble and Keeble 1904). And, when they are exposed to acidic conditions, their behaviors are changed, and their symbiotic algae are expulsed rapidly (Dupont et al. 2012). *I. pulchra* respond to noxious stimuli (Tyler and Rieger 1999). The *PKD* genes of *P. naikaiensis* may be required for such behaviors of them. In particular, PKD1-2, PKD1-3, and PKD1-4 were expressed considerably more on the anterior tip of *P. naikaiensis*, which is useful to migrate to favorable circumstances because acoel flatworms only move forward by ciliary movement and change direction by bending the worm body (Tyler and Rieger 1999). *PKD1-4* was also expressed around the mouth where there are also epidermal sensory receptors (Todt and Tyler 2007), suggesting this gene is required for prey detection and feeding because acoel flatworms feed on small marine crustaceans and protists including unicellular algae for symbiosis (Brusca et al. 2016; Jennings 1957). To examine the association between acoel *PKD* genes and their behavioral repertoires, future studies are needed with RNA interference and CRISPR CAS9 genome editing.

Some of the *PKD* genes showed much larger expression than would be expected from the sensory receptors, suggesting that they were expressed in various other cell types. We observed *PKD* gene-positive cells in the region of testes. Therefore, further roles of *P. naikaiensis PKD* genes were hypothesized to be involved in the reproductive system. In *Drosophila*, a *PKD2* homolog is localized in sperm tails and is essential for transferring sperm from the uterus to sperm storage organs of the female flies (Gao 2003; Watnick et al. 2003). Acoel flatworms are hermaphroditic and reproduce by internal fertilization. Sperms are exchanged mutually and stored at the seminal bursa until the eggs are ready to fertilize (Achatz et al. 2013; Zabotin and Evtugyn 2021; Zabotin and Golubev 2014). In this study, we did not consider the maturation stage of *P. naikaiensis*. However, it was suggested that acoel *PKD* genes were also required for sperm function and fertilization. Future studies are required to compare among the expression patterns of the *PKD* genes during maturation.

## Conclusions

*P. naikaiensis* possessed epidermal sensory receptors on the entire body. To visualize the sensory receptors three-dimensionally, α-tubulin antibody and fluorescent phalloidin could be appropriate markers. We were able to reveal that the distribution of collared receptors was different between the dorsal and ventral sides of worms and that these receptors were innervated. Additionally, we identified five polycystin family genes from the *P. naikaiensis* genome. These genes were implied to be expressed in the epidermal sensory receptors, suggesting that PKDs could be appropriate specific markers for acoel epidermal sensory receptors. Further studies are needed to demonstrate the localization with their specific antibodies since our results have not shown direct evidence to localize PKDs to the sensory receptors that we observed with electron and confocal microscopies. These sensory receptors may convert mechanical and/or chemical stimuli from the environment into electrical signals via PKD channels and release neurotransmitters to efferent neurons. We have discovered additional *TRP* family genes from the *P. naikaiensis* genome in addition to *PKD* genes including *TRPP* genes (data not shown). Acoel flatworms exhibit various behavioral repertoires such as geotaxis, phototaxis, and rheotaxis. In *I. pulchra*, some *TRP* genes are expressed in the anterior tip of the worm and in the periphery of the brain (Duruz et al. 2021). Therefore, we assumed TRP channels require for those behaviors. Since Acoela belong to the phylum Xenacoelomorpha which forms a sister group to Nephrozoa or Ambulacraria, they could be a unique model for investigating the key processes that allow bilaterians to sense environmental cues.

## Data Availability

All data generated or analyzed during this study are included in this published article.

## References

[CR1] Achatz JG, Martinez P (2012). The nervous system of *Isodiametra pulchra* (Acoela) with a discussion on the neuroanatomy of the Xenacoelomorpha and its evolutionary implications. Front Zool.

[CR2] Achatz JG, Chiodin M, Salvenmoser W, Tyler S, Martinez P (2013). The Acoela: on their kind and kinships, especially with nemertodermatids and xenoturbellids (Bilateria incertae sedis). Org Divers Evol.

[CR3] Arboleda E, Hartenstein V, Martinez P, Reichert H, Sen S, Sprecher S, Bailly X (2018). An emerging system to study photosymbiosis, brain regeneration, chronobiology, and behavior: the marine Acoel *Symsagittifera roscofensis*. BioEssays.

[CR4] Arimoto A, Hikosaka-Katayama T, Hikosaka A, Tagawa K, Inoue T, Ueki T, Yoshida M, Kanda M, Shoguchi E, Hisata K, Satoh N (2019) A draft nuclear-genome assembly of the acoel flatworm *Praesagittifera naikaiensis*. GigaScience 8:giz023. 10.1093/gigascience/giz023 10.1093/gigascience/giz023 PMC6451197 30953569

[CR5] Barr MM (2005). *Caenorhabditis elegans* as a model to study renal development and disease: sexy cilia. J Am Soc Nephrol.

[CR6] Barr MM, Sternberg PW (1999). A polycystic kidney-disease gene homologue required for male mating behaviour in *C. elegans*. Nature.

[CR7] Bedini C, Lanfranchi A (1991). The central and peripheral nervous system of Acoela (Plathelminthes). An Electron Microscopical Study Acta Zool.

[CR8] Bedini C, Ferrero E, Lanfranchi A (1973). The ultrastructure of ciliary sensory cells in two Turbellaria Acoela. Tissue Cell.

[CR9] Bery A, Cardona A, Martinez P, Hartenstein V (2010). Structure of the central nervous system of a juvenile acoel, *Symsagittifera roscoffensis*. Dev Genes Evol.

[CR10] Bezares-Calderón LA, Berger J, Jékely G (2020). Diversity of cilia-based mechanosensory systems and their functions in marine animal behaviour. Phil Trans R Soc B.

[CR11] Brusca RC, Moore W, Shuster SM (2016). Invertebrates.

[CR12] Cannon JT, Vellutini BC, Smith J, Ronquist F, Jondelius U, Hejnol A (2016). Xenacoelomorpha is the sister group to Nephrozoa. Nature.

[CR13] Cribb B, Chisholm L, Gould R, Whittington I (2003). Morphology, ultrastructure, and implied function of ciliated sensory structures on the developmental stages of *Merizocotyle icopae* (Monogenea: Monocotylidae). Microsc Res Tech.

[CR14] Dupont S, Moya A, Bailly X (2012). Stable photosymbiotic relationship under CO_2_-induced acidification in the acoel worm *Symsagittifera roscoffensis*. PLoS ONE.

[CR15] Duruz J, Kaltenrieder C, Ladurner P, Bruggmann R, Martìnez P, Sprecher SG (2021). Acoel single-cell transcriptomics: cell type analysis of a deep branching bilaterian. Mol Biol Evol.

[CR16] Eddy SR (1998). Profile hidden Markov models. Bioinformatics.

[CR17] Esarte Palomero O, Larmore M, DeCaen PG (2023). Polycystin channel complexes. Annu Rev Physiol.

[CR18] Ferrero E (1973). A fine structural analysis of the statocyst in Turbellaria Acoela. Zool Scr.

[CR19] Finn RD, Coggill P, Eberhardt RY, Eddy SR, Mistry J, Mitchell AL, Potter SC, Punta M, Qureshi M, Sangrador-Vegas A, Salazar GA, Tate J, Bateman A (2016). The Pfam protein families database: towards a more sustainable future. Nucleic Acids Res.

[CR20] Gamble FW, Keeble F (1904). The bionomics of *Convoluta roscoffensis*, with special reference to its green cells. Proc R Soc Lond.

[CR21] Gao Z, Ruden DM, Lu X (2003). PKD2 cation channel is required for directional sperm movement and male fertility. Curr Biol.

[CR22] Hall TA (1999). BioEdit: a user-friendly biological sequence alignment editor and analysis program for Windows 95/98/NT. Nucleic Acids Symp Ser.

[CR23] Hikosaka-Katayama T, Watanuki N, Niiho S, Hikosaka A (2020). Geographical distribution and genetic diversity of *Praesagittifera naikaiensis* (Acoelomorpha) in the Seto Inland Sea, Japan. Zool Sci.

[CR24] Hulett RE, Kimura JO, Bolaños DM, Luo YJ, Ricci L, Srivastava M (2022) Acoel single-cell atlas reveals expression dynamics and heterogeneity of a pluripotent stem cell population. BioRxiv 2022–02. 10.1101/2022.02.10.479464 10.1038/s41467-023-38016-4 PMC10163032 37147314

[CR25] Jennings JB (1957). Studies on feeding, digestion, and food storage in free-living flatworms (Platyhelminthes: Turbellaria). Biol Bull.

[CR26] Jondelius U, Wallberg A, Hooge M, Raikova OI (2011). How the worm got its pharynx: phylogeny, classification and Bayesian assessment of character evolution in Acoela. Syst Biol.

[CR27] Keeble F (1912). Plant-animals: a study in symbiosis.

[CR28] Köttgen M, Buchholz B, Garcia-Gonzalez MA, Kotsis F, Fu Z, Doerken M, Boehlke C, Steffl D, Tauber R, Wegierski T, Nitschke R, Suzuki M, Kramer-Zucker A, Germino GG, Watnick T, Prenen J, Nilius B, Kuehn EW, Walz G (2008). TRPP2 and TRPV4 form a polymodal sensory channel complex. J Cell Biol.

[CR29] Larkin MA, Blackshields G, Brown NP, Chenna R, McGettigan PA, McWilliam H, Valentin F, Wallace IM, Wilm A, Lopez R, Thompson JD, Gibson TJ, Higgins DG (2007). Clustal W and Clustal X version 2.0. Bioinformatics.

[CR30] Marlétaz F, Peijnenburg KT, Goto T, Satoh N, Rokhsar DS (2019). A new spiralian phylogeny places the enigmatic arrow worms among gnathiferans. Curr Biol.

[CR31] Martinez P, Hartenstein V, Sprecher S (2017). Xenacoelomorpha nervous systems. Oxford Research Encyclopedia of Neuroscience.

[CR32] Martinez P, Hartenstein V, Gavilán B, Sprecher SG, Bailly X, Boutet A, Schierwater B (2021). *Symsagittifera roscoffensis* as a model in biology. Handbook of marine model organisms in experimental biology.

[CR33] McGrath J, Somlo S, Makova S, Tian X, Brueckner M (2003). Two populations of node monocilia initiate left-right asymmetry in the mouse. Cell.

[CR34] McLaughlin S (2017). Evidence that polycystins are involved in *Hydra* cnidocyte discharge. Invertebr Neurosci.

[CR35] Mulhair PO, McCarthy CG, Siu-Ting K, Creevey CJ, O’Connell MJ (2021) Enriching for orthologs increases support for Xenacoelomorpha and Ambulacraria sister relationship. BioRxiv 2021–12. 10.1016/j.cub.2022.10.036 36356574

[CR36] Nissen M, Shcherbakov D, Heyer A, Brummer F, Schill RO (2015). Behaviour of the plathelminth *Symsagittifera roscoffensis* under different light conditions and the consequences for the symbiotic algae *Tetraselmis convolutae*. J Exp Biol.

[CR37] Nonaka S, Shiratori H, Saijoh Y, Hamada H (2002). Determination of left-right patterning of the mouse embryo by artificial nodal flow. Nature.

[CR38] Perriere G, Gouy M (1996). WWW-query: an on-line retrieval system for biological sequence banks. Biochimie.

[CR39] Pfistermüller R, Tyler S (2002). Correlation of fluorescence and electron microscopy of F-actin-containing sensory cells in the epidermis of *Convoluta pulchra* (Platyhelminthes: Acoela). Acta Zool.

[CR40] Philippe H, Poustka AJ, Chiodin M, Hoff KJ, Dessimoz C, Tomiczek B, Schiffer PH, Müller S, Domman D, Horn M, Kuhl H, Timmermann B, Satoh N, Hikosaka-Katayama T, Nakano H, Rowe ML, Elphick MR, Thomas-Chollier M, Hankeln T, Mertes F, Wallberg A, Copley RR, Martinez P, Telford MJ (2019). Mitigating anticipated effects of systematic errors supports sister-group relationship between Xenacoelomorpha and Ambulacraria. Curr Biol.

[CR41] Praetorius HA, Spring KR (2003). Removal of the MDCK cell primary cilium abolishes flow sensing. J Membr Biol.

[CR42] Praetorius HA, Spring KR (2003). The renal cell primary cilium functions as a flow sensor. Curr Opin Nephrol Hypertens.

[CR43] Raikova OI, Reuter M, Kotikova EA, Gustafsson MK (1998). A commissural brain! The pattern of 5-HT immunoreactivity in Acoela (Plathelminthes). Zoomorphology.

[CR44] Reichmuth C, Becker S, Benz M, Debel K, Reisch D, Heimbeck G, Hofbauer A, Klagges B, Pflugfelder GO, Buchner E (1995). The sap47 gene of *Drosophila melanogaster* codes for a novel conserved neuronal protein associated with synaptic terminals. Mol Brain Res.

[CR45] Rieger RM, Tyler S, Smith JPS, Rieger GE, Harrison FW, Gardiner SL (1991). Platyhelminthes: Turbellaria. Microscopic anatomy of invertebrates.

[CR46] Sakagami T, Watanabe K, Ikeda R, Ando M (2021). Structural analysis of the statocyst and nervous system of *Praesagittifera naikaiensis*, an acoel flatworm, during development after hatching. Zoomorphology.

[CR47] Semmler H, Bailly X, Wanninger A (2008). Myogenesis in the basal bilaterian *Symsagittifera roscoffensis* (Acoela). Front Zool.

[CR48] Sprecher SG, Bernardo-Garcia FJ, van Giesen L, Hartenstein V, Reichert H, Neves R, Bailly X, Martinez P, Brauchle M (2015). Functional brain regeneration in the acoel worm *Symsagittifera roscoffensis*. Biol Open.

[CR49] Todt C, Tyler S (2007). Ciliary receptors associated with the mouth and pharynx of Acoela (Acoelomorpha): a comparative ultrastructural study. Acta Zool.

[CR50] Tyler S, Bereiter-Hahn J, Matoltsy S, Richards KS (1984). Turbellarian platyhelminths. Biology of the integument.

[CR51] Tyler S, Rieger RM (1999). Functional morphology of musculature in the acoelomate worm, *Convoluta pulchra* (Plathelminthes). Zoomorphology.

[CR52] Watnick TJ, Jin Y, Matunis E, Kernan MJ, Montell C (2003). A flagellar polycystin-2 homolog required for male fertility in *Drosophila*. Curr Biol.

[CR53] Watson NA, Rohde K (1994). Two new sensory receptors in *Gyrodactylus* sp. (Platyhelminthes, Monogenea, Monopisthocotylea). Parasitol Res.

[CR54] Whittington IA, Chisholm LA, Rohde K (1999). The larvae of Monogena (Platyhelminthes). Adv Parasitol.

[CR55] Yamasu T (1982). Five new species of acoel flat worms from Japan. Galaxea.

[CR56] Yamasu T (1991). Fine structure and function of ocelli and sagittocysts of acoel flatworms. Hydrobiol.

[CR57] Zabotin YI (2019) Ultrastructure of epidermal sensillae in three species of Acoela. Invert Zool 16:71–77. 10.15298/invertzool.16.1.08

[CR58] Zabotin YI, Evtugyn VG (2021). Ultrastructure of spermatozoa and female copulatory organs in preferably asexually-reproducing acoel *Convolutriloba retrogemma* (Acoelomorpha). Zoomorphology.

[CR59] Zabotin YI, Golubev AI (2014). Ultrastructure of oocytes and female copulatory organs of Acoela. Biol Bull Russ Acad Sci.

